# Insights Into the Epidemiological, Clinical, Histopathological, and Dermoscopic Aspects of Chronic Plaque Psoriasis

**DOI:** 10.7759/cureus.69912

**Published:** 2024-09-22

**Authors:** Rishabh Jain, Ravi Sangoi, Shoraf Pascal, Yagyavalkya Sharma, Yash Variya Takodara, Amit Ajay Singh, Yashvee Shah, Prashanth A, Mohit Agrawal, Pratibha Wankhede

**Affiliations:** 1 General Medicine, Jagadguru Sri Shivarathreeshwara Academy of Higher Education and Research, Mysore, IND; 2 Internal Medicine, Punyashlok Ahilyadevi Holkar Government Medical College and General Hospital, Baramati, IND; 3 Community Medicine, Madha Medical College and Research Institute, Chennai, IND; 4 Life Sciences, Team MP Research Work, Mathura, IND; 5 Biotechnology, Kalp Laboratories, Mathura, IND; 6 Internal Medicine, Government Medical College, Alibag, IND; 7 Internal Medicine, Government Medical College, Latur, IND; 8 Global Health, New York University School of Global Public Health, New York, USA; 9 Physiology, Mahatma Gandhi Institute of Medical Sciences, Wardha, IND; 10 Physical Medicine and Rehabilitation, S.S. Agrawal Institute of Physiotherapy and Medical Care Education, Navsari, IND; 11 Public Health, Shalinitai Meghe College of Nursing, Datta Meghe Institute of Higher Education and Research, Wardha, IND

**Keywords:** chronic plaque psoriasis, clinical features, dermoscopy, epidemiology, histopathology

## Abstract

Background

Psoriasis is a persistent inflammatory condition of the skin marked by clearly demarcated red plaques adorned with silvery scales. It impacts individuals across different age ranges and presents with unique clinical, histological, and dermoscopic characteristics. This research seeks to offer an extensive assessment of the demographic, clinical, histological, and dermoscopic attributes of chronic plaque psoriasis (CPP).

Methodology

A total of 60 patients with CPP were included in this study. Data were collected on demographic characteristics, disease duration, clinical features, histopathological findings, and dermoscopic patterns. Statistical analysis was performed using IBM SPSS Statistics for Windows, Version 20 (Released 2011; IBM Corp., Armonk, New York), employing descriptive statistics to summarize demographic and clinical characteristics and histopathological and dermoscopic findings.

Results

The study included 60 patients with CPP, predominantly aged 41-50 years (n=18, 30%), with a higher prevalence in males (n=35, 58.3%). Plaque psoriasis was the most common type observed (75%), with lesions primarily located on the scalp (n=30, 50%) and elbows (n=25, 41.7%), consistent with typical psoriasis distribution patterns. Histopathological analysis revealed acanthosis in 55 patients (91.7%) and parakeratosis in 50 patients (83.3%), indicating thickened epidermis and retention of nuclei in the stratum corneum, which are characteristic of psoriasis. Additionally, Munro's microabscesses were found in 30 patients (50%) and spongiform pustules in 10 patients (16.7%), supporting the diagnosis through classic markers. Dermoscopic evaluations identified red dots or globules in 55 patients (91.7%) and white scales in 50 patients (83.3%), essential for differentiating psoriasis from other skin conditions. Further dermoscopic findings included micro-erosions in 25 patients (41.7%), hemorrhagic spots in 15 patients (25%), and yellowish scales in 20 patients (33.3%), reflecting disease activity and inflammation.

Conclusion

This study underscores the importance of a multifaceted approach in diagnosing and managing CPP. The prevalence of psoriasis in middle-aged males and the common clinical presentation on the scalp and elbows are consistent with previous studies. Histopathological and dermoscopic features provide critical diagnostic support and can guide effective treatment strategies. Continued research is essential to enhance understanding and management of this prevalent dermatological condition.

## Introduction

Psoriasis is a chronic, inflammatory, and proliferative skin disorder marked by distinct elevated red patches and plaques topped with silvery-white scales. This condition varies significantly in terms of duration, severity, and clinical morphology, which contributes to its complex management. Chronic plaque psoriasis (CPP) is identified as the most frequent form of psoriasis, usually appearing as clearly defined, reddish, flaky patches that can range in size from small patches to large areas of involvement [1]. The prevalence of psoriasis differs widely across various populations globally, with estimates ranging from 0% to 11.8%. In the Indian population, the prevalence is reported to be between 0.44% and 2.8%, highlighting its significant impact on public health in diverse regions [2]. Among the key histopathological features essential for diagnosing psoriasis are the spongiform pustule and Munro microabscess, which are distinctive markers of the disease [3].

Dermoscopy, a non-invasive diagnostic tool, has gained prominence in dermatology for its ability to reveal morphologic features of skin lesions that are not visible to the naked eye. This tool serves as an intermediary between traditional clinical dermatology and microscopic dermatopathology, offering enhanced accuracy in diagnosing skin conditions. In particular, dermoscopy has been instrumental in identifying specific patterns in various inflammatory skin diseases. It has proven to be highly effective in distinguishing psoriasis from other common inflammatory disorders such as lichen planus [4,5].

In cases of CPP, dermoscopic examination typically displays evenly spread-out speckled blood vessels across the affected area, contrasted with a pale red backdrop. This vascular pattern, accompanied by diffuse white scales, is highly indicative of CPP and serves as a reliable diagnostic criterion [6,7]. The utility of dermoscopy extends beyond diagnosis, offering insights into the disease's progression and response to treatment, making it an invaluable tool in the clinical setting [8]. This study is designed to explore and elucidate the demographic, clinical, dermoscopic, and histological characteristics of individuals diagnosed with CPP. By providing a comprehensive analysis, the research aims to enhance understanding of the disease's manifestations and improve diagnostic accuracy and patient outcomes.

## Materials and methods

Study design and setting

This prospective, cross-sectional study was conducted in the dermatology outpatient department over the course of one year, spanning from July 2023 to June 2024. All patients provided informed consent prior to their inclusion in the study. The study commenced after the approval of the institutional ethical committee of Madha Medical College and Research Institute (MMCRI/IEC/2023/031).

Sample Size Calculation

The sample size for this study was determined using the standard formula for calculating sample size in cross-sectional studies: n=Z2∙p∙q/E2. Here, Z represents the Z-value corresponding to the desired confidence level, which was set at 1.96 for a 95% confidence interval. The prevalence (p) of CPP was estimated at 5%, with (q) representing the complement of p, i.e., (1 - p). The margin of error (E) was set at 0.05. Using these parameters, the sample size was calculated to be 60 patients, ensuring adequate power to detect significant associations within the study.

Selection Criteria

The study's selection criteria included patients diagnosed with psoriasis who consented to a written, informed agreement for participation. Patients were excluded if they were under 18 years of age, diagnosed with malignancies or other immunocompromised conditions, or pregnant.

Data sources and variables

A comprehensive medical history was meticulously gathered for each patient, ensuring that all relevant factors, including the duration and progression of the disease, were thoroughly documented. Following this, an exhaustive examination was conducted to assess the condition of the skin, mucous membranes, and hair, focusing on identifying any visible abnormalities or lesions. The assessment was carried out with precision to ensure that no clinical signs were overlooked. Dermoscopic evaluations were then performed on all cutaneous lesions using the advanced DermLite DL3 device (Aliso Viejo, California), which provided enhanced visualization of the sub-surface skin structures. Each lesion was meticulously photographed using a high-resolution Canon Ixus camera (Tokyo, Japan), ensuring that the images captured were clear and suitable for further analysis. To address potential inter-observer variability in dermoscopic evaluations, all images were reviewed by multiple trained dermatologists. Each dermatologist independently assessed the images and recorded their findings. The results were then compared, and discrepancies were resolved through consensus meetings. This approach ensured that the interpretation of dermoscopic features was consistent and reliable across different observers.

In addition to the clinical examinations, baseline laboratory investigations were conducted, including a complete blood count to assess the patient's overall health, liver function tests to rule out hepatic involvement, and renal function tests to evaluate kidney function. These tests were crucial in identifying any underlying systemic conditions that could influence the patient's treatment plan. Furthermore, skin and mucosal biopsies were obtained from the affected areas, providing a tissue sample for histopathological examination. This biopsy process involved carefully excising a small portion of the lesion under sterile conditions. The collected tissue samples were then processed and analyzed by expert pathologists. To manage inter-observer variability in histopathological analysis, multiple pathologists independently examined the tissue samples. Their findings were cross-checked, and any differences in interpretations were discussed in review meetings to reach a consensus. This ensured that the histopathological diagnoses were accurate and consistent. The pathologists' detailed reports played a critical role in guiding the clinical management and treatment strategies for each patient.

Statistical analysis

Descriptive statistics were used to analyze the data. Frequencies and percentages were calculated for categorical variables. Data presentation involved summarizing the results in tables, with counts and percentages provided for each category. Statistical analysis was performed using IBM SPSS Statistics for Windows, Version 20 (Released 2011; IBM Corp., Armonk, New York).

## Results

Table 1 describes the demographic characteristics of the study participants. Among the 60 participants, 12 (20%) were aged 18-30 years, 15 (25%) were aged 31-40 years, 18 (30%) were aged 41-50 years, 10 (16.7%) were aged 51-60 years, and five (8.3%) were over 60 years. The gender distribution was 35 males (58.3%) and 25 females (41.7%). In terms of the duration of psoriasis, 12 participants (20%) had psoriasis for less than three months, 15 (25%) had it for three to six months, 18 (30%) had it for six to nine months, and 15 (25%) had it for 9-12 months. Additionally, 22 participants (36.7%) had comorbidities, while 38 (63.3%) did not.

**Table 1:** Demographic characteristics of the study participants

Characteristic	Category	Count (N=60)	Percentage (%)
Age	18-30 years	12	20
	31-40 years	15	25
	41-50 years	18	30
	51-60 years	10	16.7
	>60 years	5	8.3
Gender	Male	35	58.3
	Female	25	41.7
Duration of psoriasis	<3 months	12	20
	3-6 months	15	25
	6-9 months	18	30
	9-12 months	15	25
Presence of comorbidities	Yes	22	36.7
	No	38	63.3

**Figure 1:**
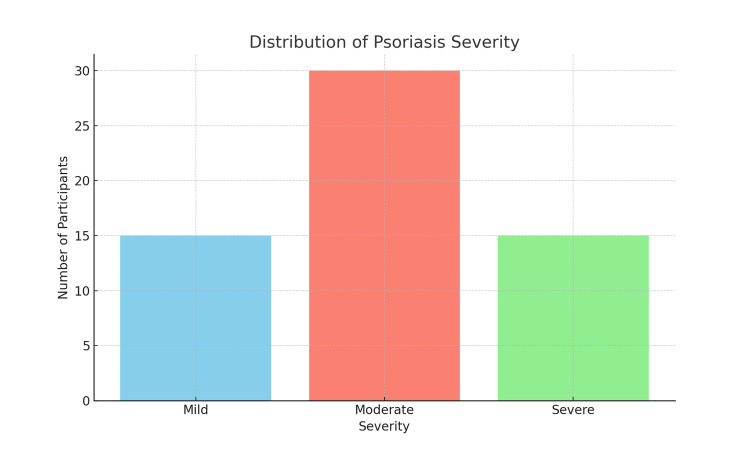
Psoriasis severity bar graph

Table 2 describes the clinical features of psoriasis among the study participants. Plaque psoriasis was observed in 45 participants (75%). Guttate psoriasis was found in five (8.3%), inverse psoriasis in four (6.7%), pustular psoriasis in three (5%), and erythrodermic psoriasis in three (5%). The lesions were located on the scalp in 30 participants (50%), elbows in 25 (41.7%), knees in 20 (33.3%), trunk in 18 (30%), and nails in eight (13.3%). The severity of psoriasis was classified as mild in 15 participants (25%), moderate in 30 (50%), and severe in 15 (25%). The duration of psoriasis was categorized as less than three months for 12 participants (20%), three to six months for 15 (25%), six to nine months for 18 (30%), and 9-12 months for 15 (25%). Figure 1 presents the distribution of psoriasis severity in a bar graph format, illustrating the proportion of participants with mild, moderate, and severe psoriasis.

**Table 2:** Clinical features of psoriasis

Clinical Feature	Category	Count (N=60)	Percentage (%)
Type of psoriasis	Plaque	45	75
	Guttate	5	8.3
	Inverse	4	6.7
	Pustular	3	5
	Erythrodermic	3	5
Location of lesions	Scalp	30	50
	Elbows	25	41.7
	Knees	20	33.3
	Trunk	18	30
	Nails	8	13.3
Severity	Mild	15	25
	Moderate	30	50
	Severe	15	25
Duration of psoriasis	<3 months	12	20
	3-6 months	15	25
	6-9 months	18	30
	9-12 months	15	25

Table 3 describes the histopathological features observed in the study. Parakeratosis was present in 50 samples (83.3%), acanthosis in 55 (91.7%), and hyperkeratosis in 45 (75%). Munro's microabscesses were noted in 30 samples (50%), spongiform pustules in 10 (16.7%), and dermal papillary dilatation in 40 (66.7%).

**Table 3:** Histopathological feature

Histopathological Feature	Count (N=60)	Percentage (%)
Parakeratosis	50	83.3
Acanthosis	55	91.7
Hyperkeratosis	45	75
Munro’s microabscesses	30	50
Spongiform pustules	10	16.7
Dermal papillary dilatation	40	66.7

Table 4 describes the dermoscopic features identified in the study. Red dots or globules were observed in 55 samples (91.7%), white scales in 50 (83.3%), and micro-erosions in 25 (41.7%). Hemorrhagic spots were found in 15 samples (25%), and yellowish scales were present in 20 (33.3%).

**Table 4:** Dermoscopic feature

Dermoscopic Feature	Count (N=60)	Percentage (%)
Red dots/globules	55	91.7
White scales	50	83.3
Micro-erosions	25	41.7
Hemorrhagic spots	15	25
Yellowish scale	20	33.3

## Discussion

This research offers an in-depth exploration of CPP, focusing on various aspects of the condition. The findings provide valuable insights into the prevalence, clinical manifestations, and diagnostic characteristics of this dermatological condition. The study included 60 patients with CPP, with a notable prevalence in the 41-50 years age group (18 patients, 30%). This is consistent with existing literature that identifies psoriasis as more common among middle-aged individuals [1,2]. The gender distribution revealed a higher prevalence in males (35 patients, 58.3%) compared to females (25 patients, 41.7%), which aligns with previous studies indicating a male predominance, although the difference is not always significant [9-11].

The duration of psoriasis varied among participants, with the highest proportion having the condition for six to nine months (18 patients, 30%) or 9-12 months (15 patients, 25%). This reflects the chronic nature of psoriasis and underscores the ongoing need for effective management strategies [12,13]. The findings highlight the substantial burden of long-term psoriasis management. The clinical features revealed that plaque psoriasis was the most common type, affecting 45 patients (75%). This finding corroborates previous research indicating that plaque psoriasis is the predominant form of the disease [3,14]. Lesions were most frequently located on the scalp (30 patients, 50%) and elbows (25 patients, 41.7%), consistent with the typical distribution patterns observed in psoriasis [15]. The severity classification showed mild psoriasis in 15 patients (25%), moderate psoriasis in 30 patients (50%), and severe psoriasis in 15 patients (25%), demonstrating a range of disease severity [16].

The histopathological analysis identified acanthosis in 55 patients (91.7%) and parakeratosis in 50 patients (83.3%), which are characteristic of psoriasis [3,7]. Munro's microabscesses were observed in 30 patients (50%), while spongiform pustules were found in 10 patients (16.7%). These findings align with established histopathological markers of psoriasis and provide crucial diagnostic information [6]. The combination of these findings enhances diagnostic specificity by distinguishing psoriasis from other dermatological conditions, such as eczema or lichen planus, where overlapping but incomplete features may occur. Dermal papillary dilatation was present in 40 patients (66.7%), further supporting the diagnosis of psoriasis and differentiating it from other skin conditions.

The dermoscopic evaluation revealed red dots or globules in 55 patients (91.7%) and white scales in 50 patients (83.3%). These features are essential for the dermoscopic diagnosis of psoriasis, as supported by previous research [4,5]. The combined presence of red dots and white scales enhances diagnostic accuracy for CPP compared to similar conditions, such as seborrheic dermatitis, where such patterns may be less pronounced or absent. Micro-erosions were observed in 25 patients (41.7%), hemorrhagic spots in 15 patients (25%), and yellowish scales in 20 patients (33.3%). These additional dermoscopic findings contribute to a comprehensive view of lesion characteristics and aid in the accurate assessment of psoriasis. However, the limitations of dermoscopy must be considered, as distinguishing psoriasis from other skin conditions, such as atopic dermatitis or seborrheic dermatitis, can be challenging in atypical cases. Future studies should focus on how dermoscopic features evolve during therapy and their potential use as biomarkers for disease progression. This study contributes to the understanding of CPP by detailing its multifaceted characteristics and emphasizing the value of integrated diagnostic approaches. Further research should explore how these specific histopathological and dermoscopic findings correlate with treatment outcomes, particularly with newer biologic therapies. Additionally, larger and more diverse samples are warranted to validate these findings and enhance the generalizability of the results [17,18].

Limitations of the study

This study has several limitations. Its cross-sectional design limits the ability to draw causal inferences or track disease progression over time. The sample size of 60 patients may not fully represent the diversity of psoriasis presentations. Conducted at a single tertiary care center, the findings may not be generalizable to other settings. Selection bias may be introduced due to the single-center recruitment process, and recall bias may affect the accuracy of patient-reported data on symptom duration or treatment responses. The absence of a control group and potential recall bias in patient reporting could also affect the accuracy and comparison of results. Future studies should aim to mitigate these biases by involving multicenter designs and longitudinal follow-up to track disease progression and treatment responses more comprehensively.

## Conclusions

The study highlights the importance of a detailed approach to understanding CPP. The demographic, clinical, histopathological, and dermoscopic findings provide valuable insights into the prevalence and diagnostic features of the condition. These insights, particularly the combination of histopathological features such as acanthosis and dermoscopic findings like red dots and white scales, underline the importance of integrated diagnostic approaches for accurate assessment and differentiation from other dermatological conditions. These insights support the need for targeted management strategies that address the diverse presentations of psoriasis and emphasize the role of comprehensive evaluations in improving patient care and treatment outcomes. Future research should focus on how these diagnostic features relate to therapeutic responses, particularly with newer biologic treatments, and investigate their potential as non-invasive markers for disease progression.
